# Supplementary material for: Potential of Tyrosine Kinase Receptor TIE-1 as Novel Therapeutic Target in High-PI3K-Expressing Ovarian Cancer
Source: Cancers (Basel). 2020 Jun 26;12(6):1705. doi: 10.3390/cancers12061705 (PMC7352248; doi:10.3390/cancers12061705)
Supplement: Supplementary file 1 [file cancers-12-01705-s001.zip › supplemental data/supplemental figure 1-4 (Cancers-816921-Zhang et al).pdf]

Supplemental Figure 1

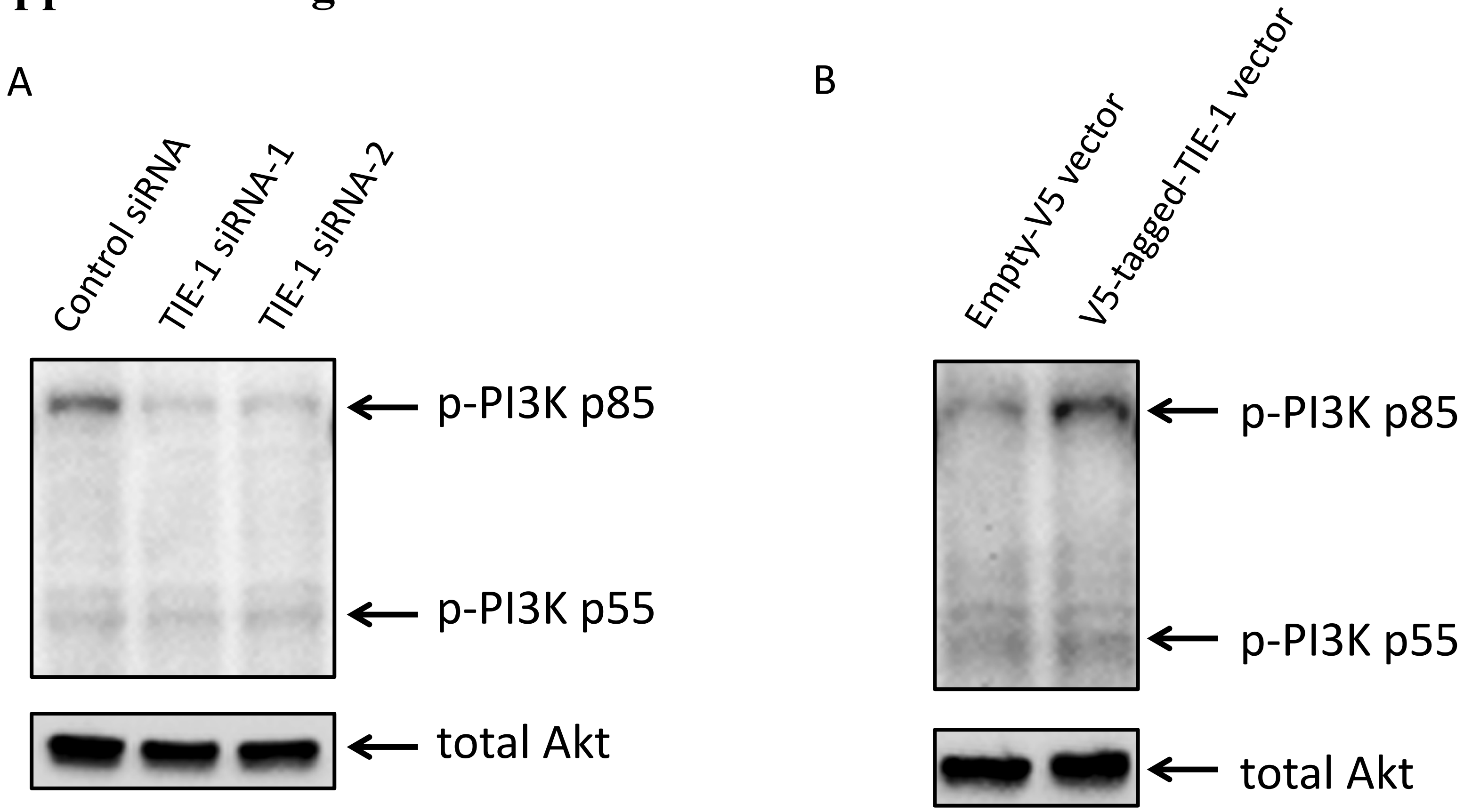

Supplemental Figure 1 TIE-1 knockdown/overexpression effect on the expression of key proteins in PI3K/Akt pathway.

A, SKOV3 ovarian cancer cells were transfected with 5 nM TIE-1 siRNA-1, TIE-1 siRNA-2, or control siRNA for 72 h. Extracted cellular proteins were subjected to immunoblot analysis with antibodies against p-PI3K and Akt. Equal amounts of proteins were loaded in each lane. Three independent experiments were performed, and representative images are shown.

B, SKOV3 ovarian cancer cells were transfected with an empty vector or V5-tagged-TIE-1 vector for 72 h. Extracted cellular proteins were subjected to immunoblot analysis with antibodies against p-PI3K and Akt. Equal amounts of proteins were loaded in each lane. Three independent experiments were performed, and representative images are shown.

Supplemental Figure 2

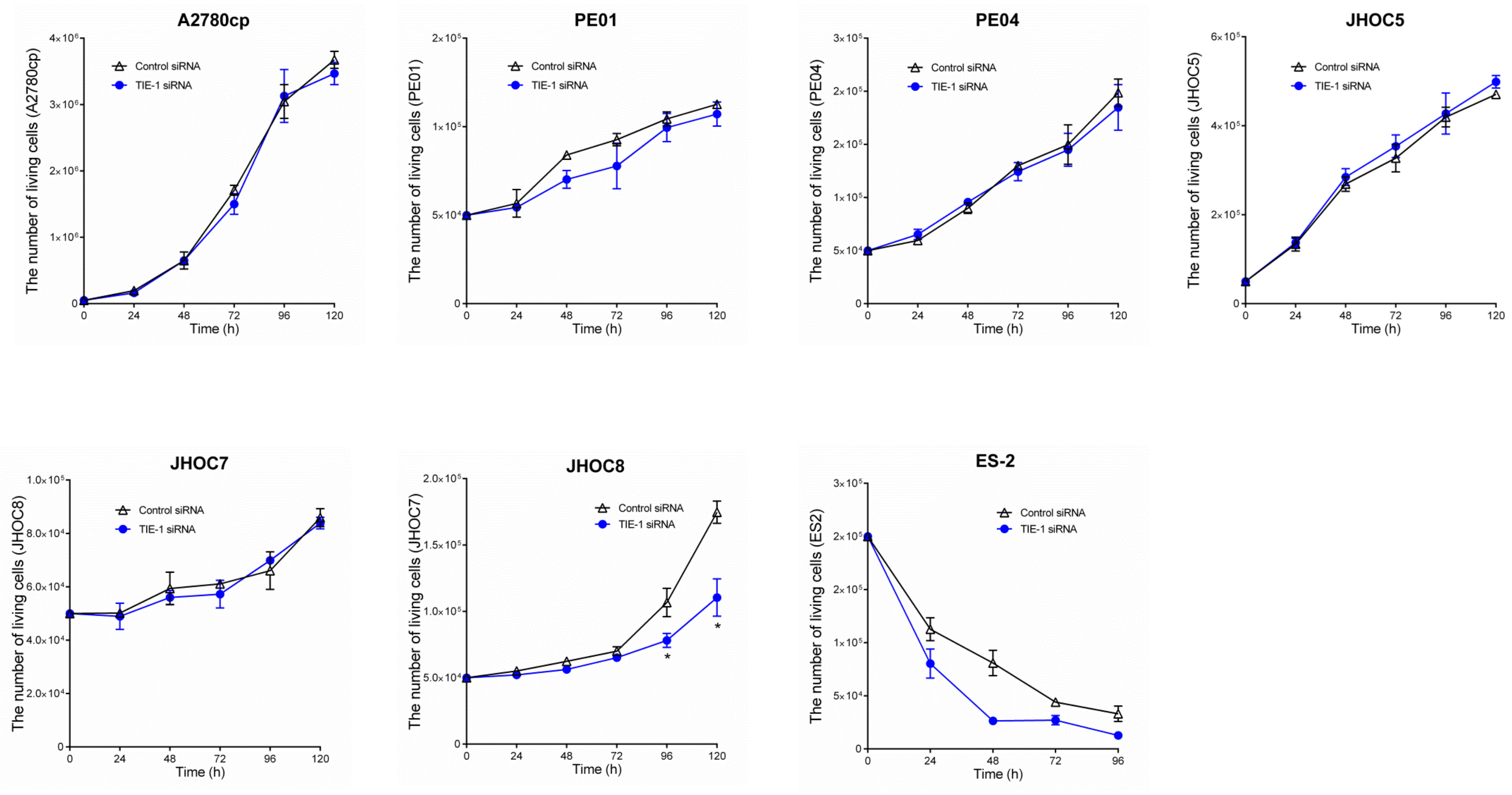

Supplemental Figure 2 The Effects of TIE-1 Knock-down on cell proliferation in multiple ovarian cancer cell lines. Seven ovarian cancer cell lines were transfected with 5 nM TIE-1 siRNA or control siRNA up to 120 h. The number of living cells was counted. Values are shown as the mean ± SD (n = 3).

Supplemental Figure 3

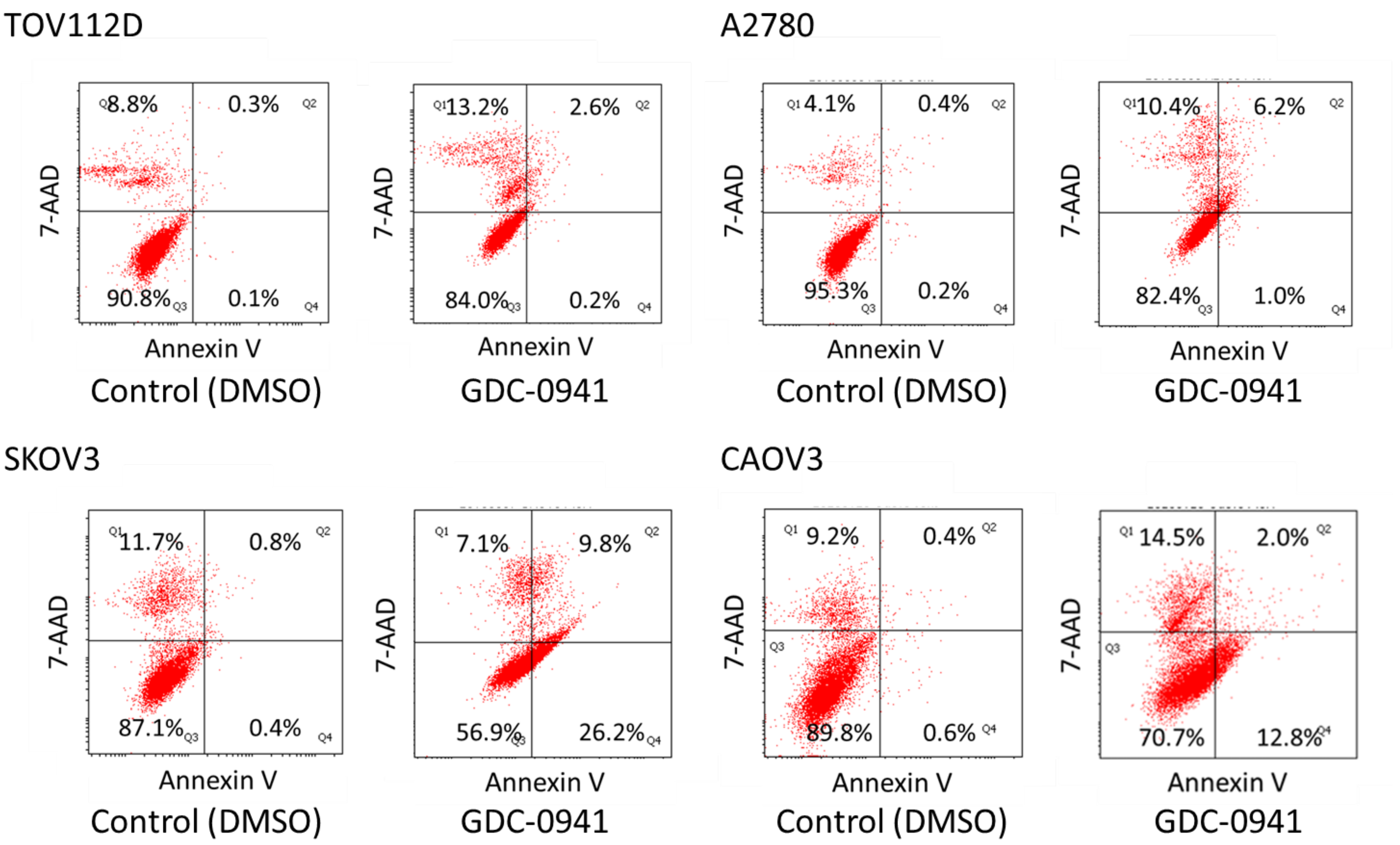

Supplemental Figure 3 PI3K inhibitor GDC-0941 induces apoptosis in multiple ovarian cancer cell lines.
Four ovarian cancer cell lines were treated with 10 μM GDC-0941 or control DMSO for 72 h and assessed as described in “Materials and Methods”. Annexin V/7-AAD assays were performed for determining apoptotic cell death.

Supplemental Figure 4

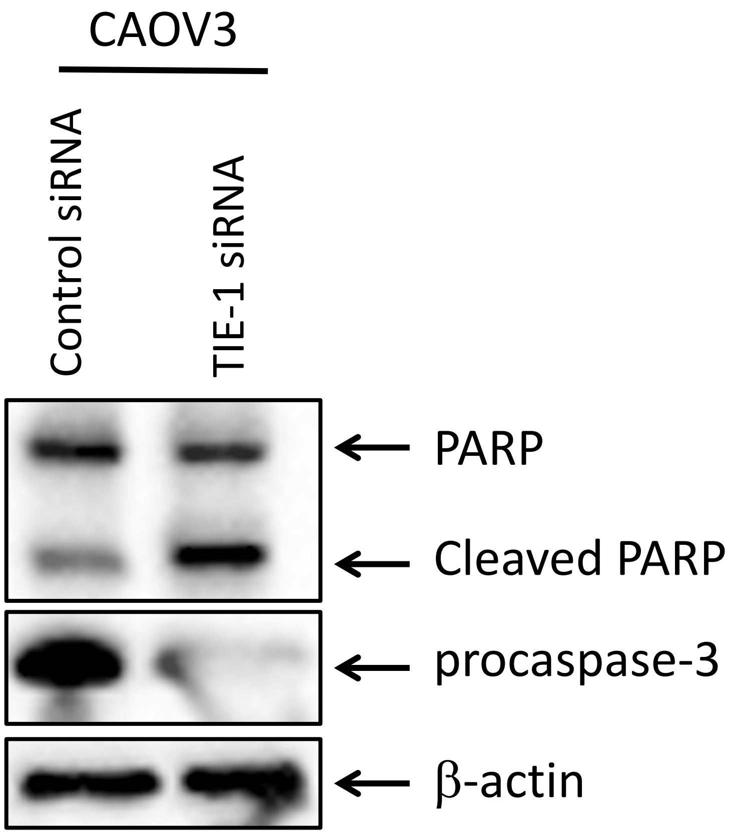

Supplemental Figure 4 Inhibition of TIE-1 induces apoptosis in PI3K high expressing CAOV3 cell lines.
CAOV3 cells were transfected with 5 nM TIE-1 siRNA or control siRNA for 72 h, and extracted cellular proteins were subjected to immunoblot analysis with antibodies for PARP, procaspase-3 and β-actin. Equal amounts of proteins were loaded in each lane. Three independent experiments were performed, and representative images are shown.
