# Supplementary material for: Potential of Tyrosine Kinase Receptor TIE-1 as Novel Therapeutic Target in High-PI3K-Expressing Ovarian Cancer
Source: Cancers (Basel). 2020 Jun 26;12(6):1705. doi: 10.3390/cancers12061705 (PMC7352248; doi:10.3390/cancers12061705)
Supplement: Supplementary file 1 [file cancers-12-01705-s001.zip › supplemental data/Supplemental data (Whole blots of western blot analysis-819621-Zhang et al).pdf]

**Supplemental Figure 5**

# Whole blots of western blot analysis

Figure 1A

TIE-1 (125 kDa)

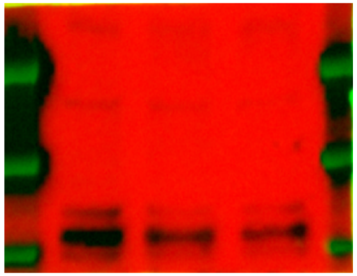


PI3K p110α (110 kDa)

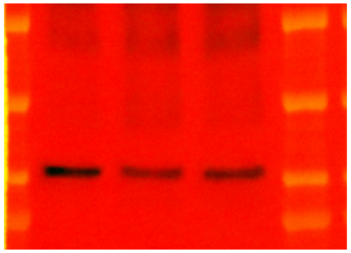


p-AKT (60 kDa)

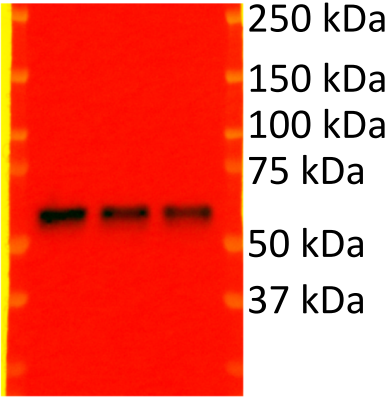


PTEN (42 kDa)

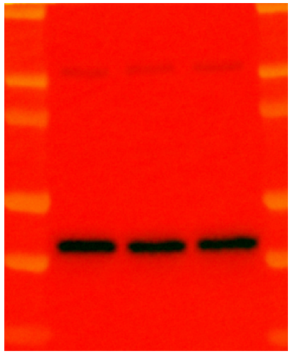


β-actin (42 kDa)

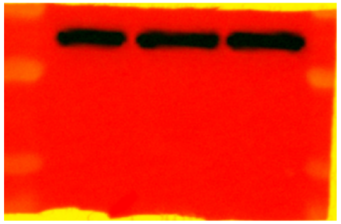


| densitometry readings | Control siRNA | TIE-1 siRNA-1 | TIE-1 siRNA-2 |
|---|---|---|---|
| TIE-1 | 81.3 | 28.8 | 35.2 |
| PI3K p110α | 30.1 | 19.1 | 22.5 |
| p-AKT | 63.6 | 49.9 | 42.5 |
| PTEN | 52.1 | 47.3 | 48.0 |
| β-actin | 63.7 | 73.0 | 73.9 |

Figure 1D

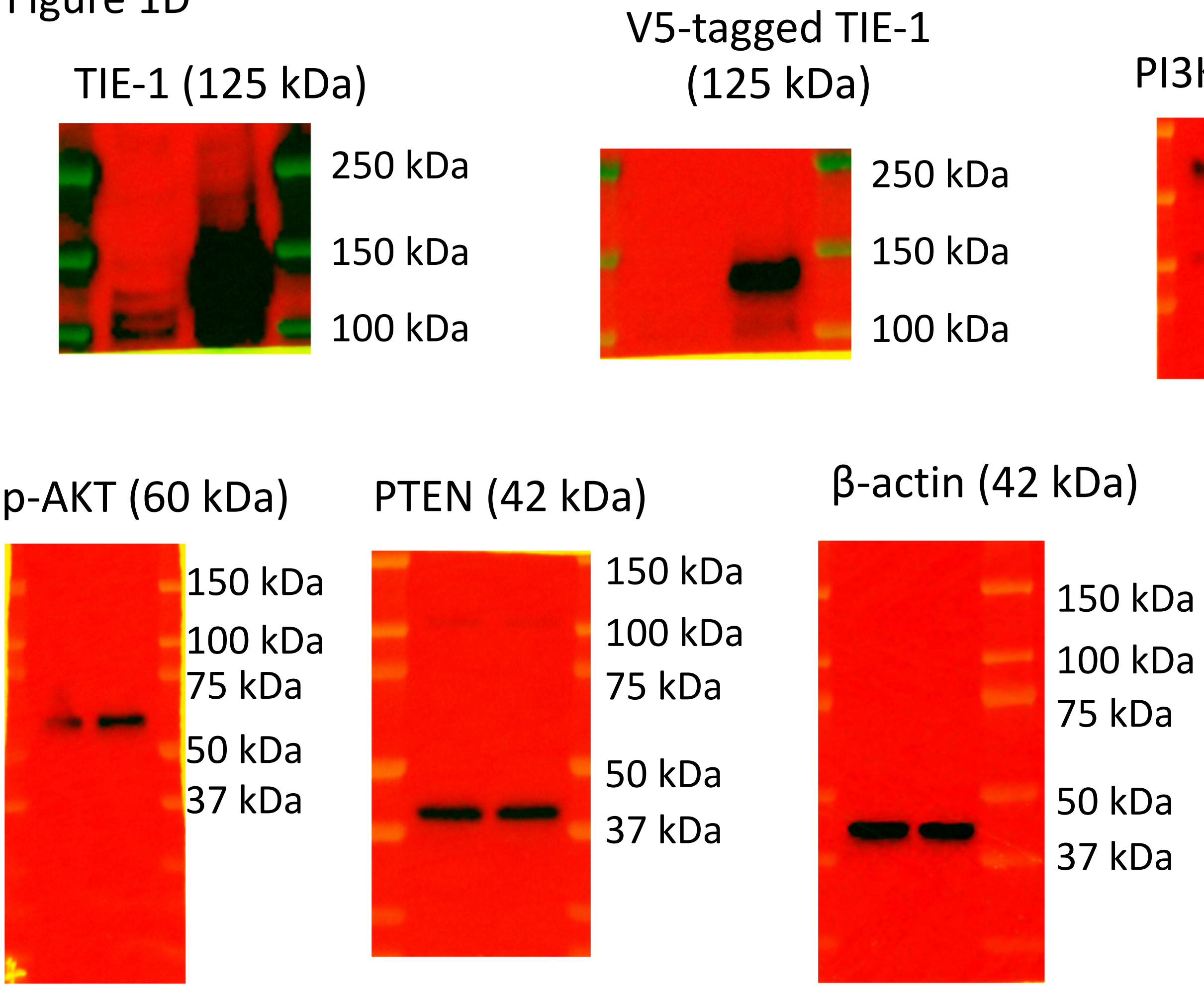


| densitometry readings | Empty vector | TIE-1-V5 vector |
|---|---|---|
| TIE-1 | 105.3 | 453.4 |
| V5-tagged TIE-1 | 5.5 | 68.3 |
| PI3K p110α | 8.1 | 42.2 |
| p-AKT | 29.7 | 57.3 |
| PTEN | 51.7 | 51.4 |
| β-actin | 25.4 | 22.3 |

Figure 2A

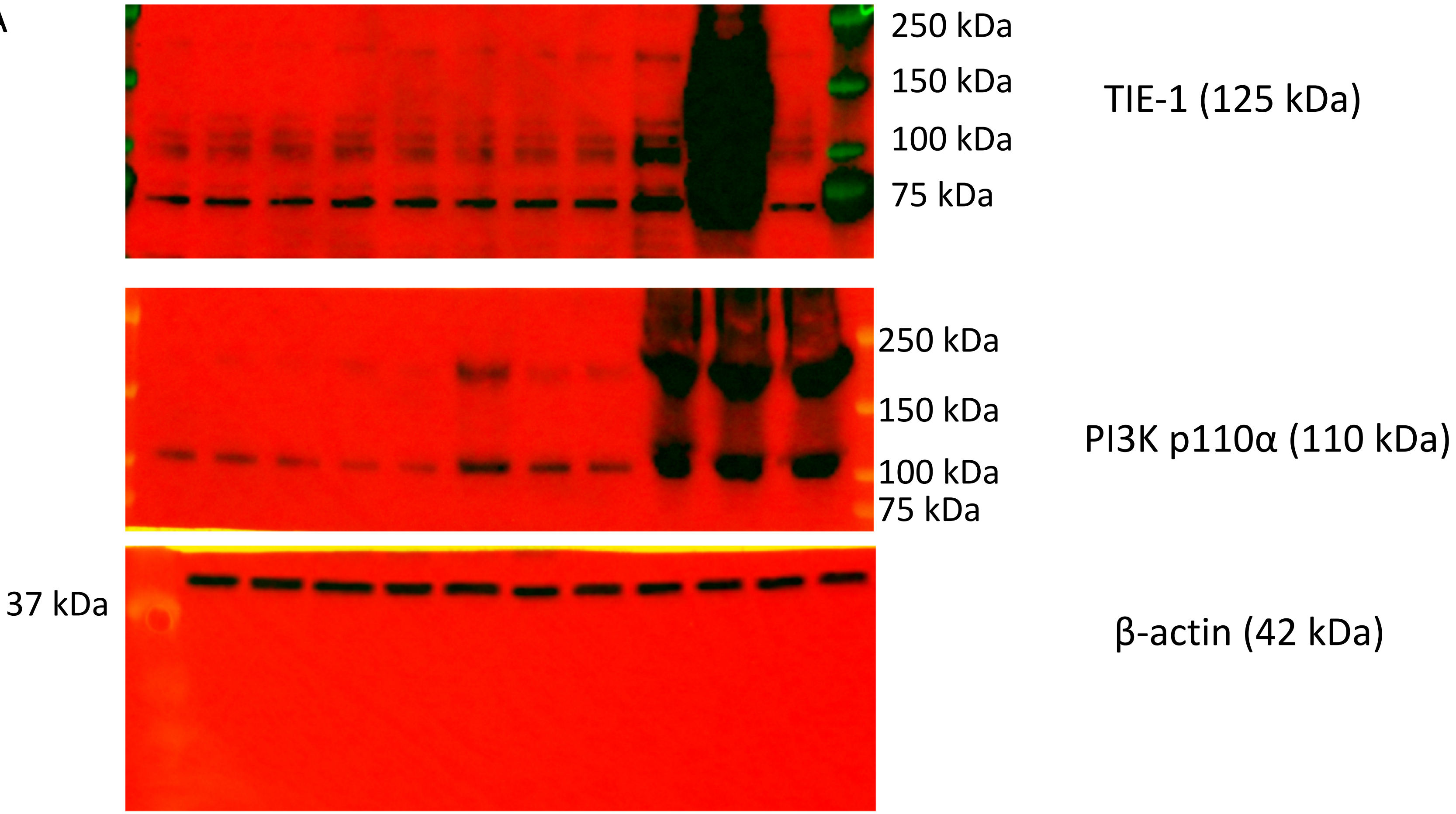


| densitometry readings | TOV112D | A2780 | A2780cp | PE01 | PE04 | CAOV3 | JHOC5 | JHOC7 | JHOC8 | ES2 | SKOV 3 |
|---|---|---|---|---|---|---|---|---|---|---|---|
| TIE-1 | 34.5 | 46.2 | 46.6 | 55.4 | 54.9 | 54.9 | 52.9 | 54.2 | 148.3 | 528.1 | 56.1 |
| PI3K p110α | 19.0 | 13.9 | 14.2 | 8.7 | 10.9 | 50.3 | 29.2 | 23.1 | 115.4 | 106.0 | 103.9 |
| β-actin | 15.3 | 14.7 | 17.0 | 13.6 | 15.5 | 13.8 | 12.5 | 12.4 | 12.6 | 13.9 | 12.5 |

Figure 2E

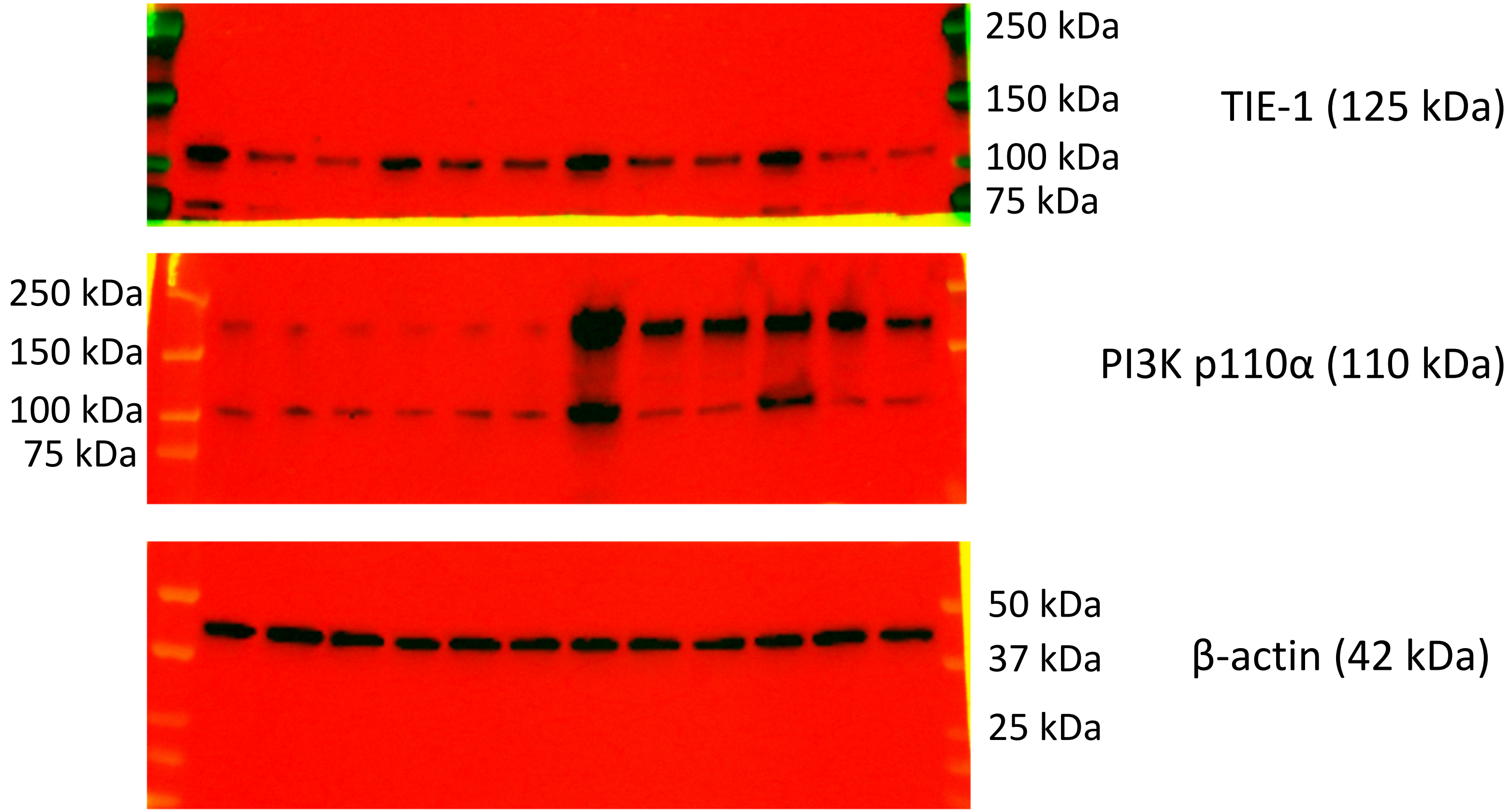


| densitometry readings | Tov con | Tov TIE-1 si-1 | Tov TIE-1 si-2 | A2780 con | A2780 TIE-1 si-1 | A2780 TIE-1 si-2 | SK con | SK TIE-1 si-1 | SK TIE-1 si-2 | CAOV 3 con | CAOV3 TIE-1 si-1 | CAOV3 TIE-1 si-2 |
|---|---|---|---|---|---|---|---|---|---|---|---|---|
| TIE-1 | 65.4 | 21.7 | 13.3 | 43.4 | 28.7 | 27.7 | 64.3 | 30.3 | 25.4 | 58.8 | 15.0 | 11.8 |
| PI3K p110α | 13.8 | 14.3 | 11.9 | 7.6 | 12.8 | 13.0 | 107.3 | 14.9 | 13.3 | 73.8 | 10.6 | 9.5 |
| β-actin | 70.4 | 84.9 | 70.9 | 53.8 | 64.5 | 60.4 | 60.1 | 61.4 | 58.1 | 64.3 | 75.4 | 62.0 |

Figure 3I

PARP (116 kDa)
Cleaved PARP (89 kDa)

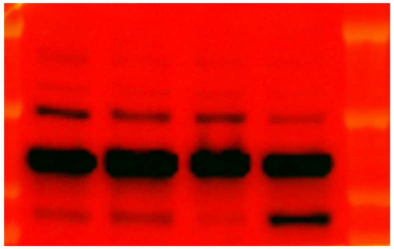


Procaspase-3 (35 kDa)

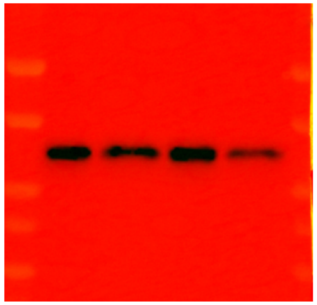


Cleaved caspase-3
(17,19 kDa)

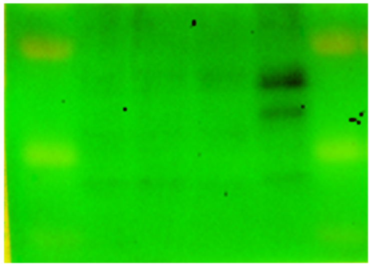


β-actin (42 kDa)

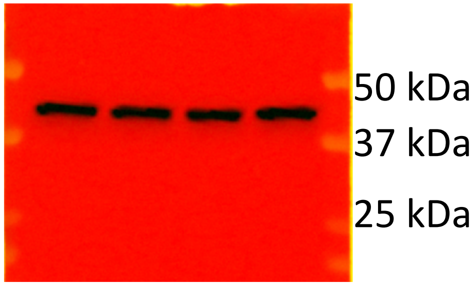


| densitometry readings | Tov112D control siRNA | Tov112D TIE-1 siRNA | SKOV3 control siRNA | SKOV3 TIE-1 siRNA |
|---|---|---|---|---|
| PARP | 117.6 | 135.4 | 114.3 | 111.7 |
| Cleaved PARP | 16.8 | 20.3 | 8.7 | 49.5 |
| Procaspase-3 | 69.2 | 64.5 | 75.9 | 36.2 |
| Cleaved caspase-3 | 64.5 | 72.5 | 81.5 | 152.5 |
| β-actin | 51.8 | 48.7 | 44.3 | 51.0 |

Figure 4A

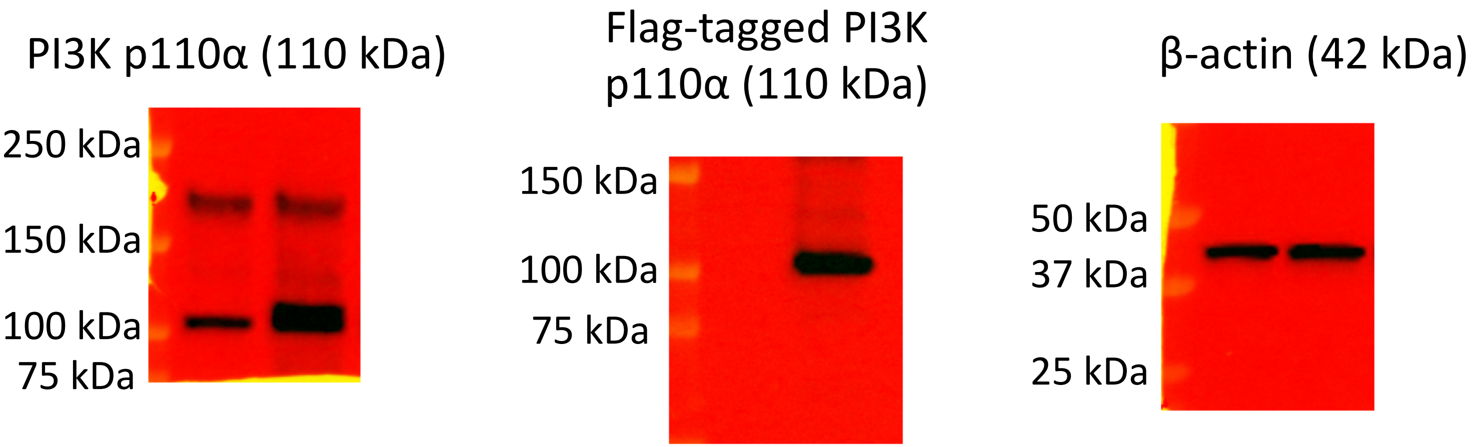


| densitometry readings | Empty vector | PI3K-Flag-vector |
|---|---|---|
| TIE-1 | 1.7 | 81.4 |
| PI3K p110α | 34.0 | 100.9 |
| β-actin | 59.7 | 68.7 |

Figure 4E

TIE-1 (125 kDa)

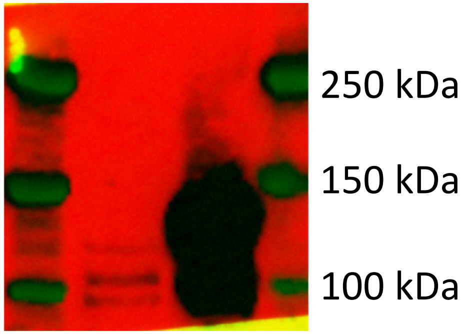


V5-tagged TIE-1
(125 kDa)

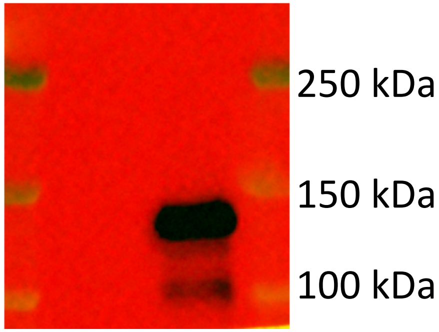


PI3K p110α (110 kDa)

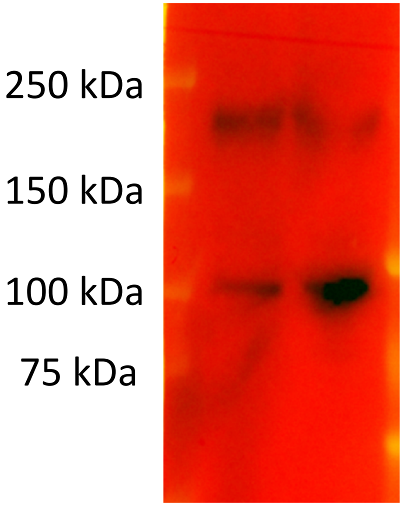


β-actin (42 kDa)

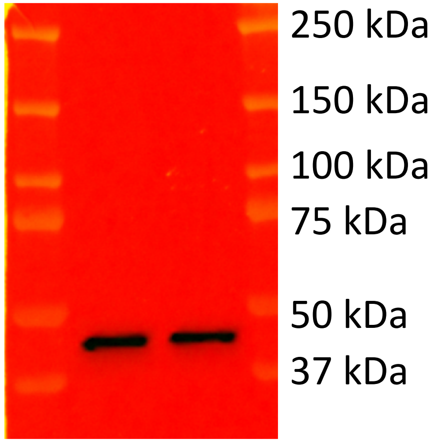


| densitometry readings | Empty vector polyclone cells | TIE-1 stably overexpressing polyclone |
|---|---|---|
| TIE-1 | 58.1 | 357.4 |
| V5-tagged TIE-1 | 5.5 | 64.1 |
| PI3K p110α | 43.1 | 81.2 |
| β-actin | 59.3 | 53.2 |

Figure 4I

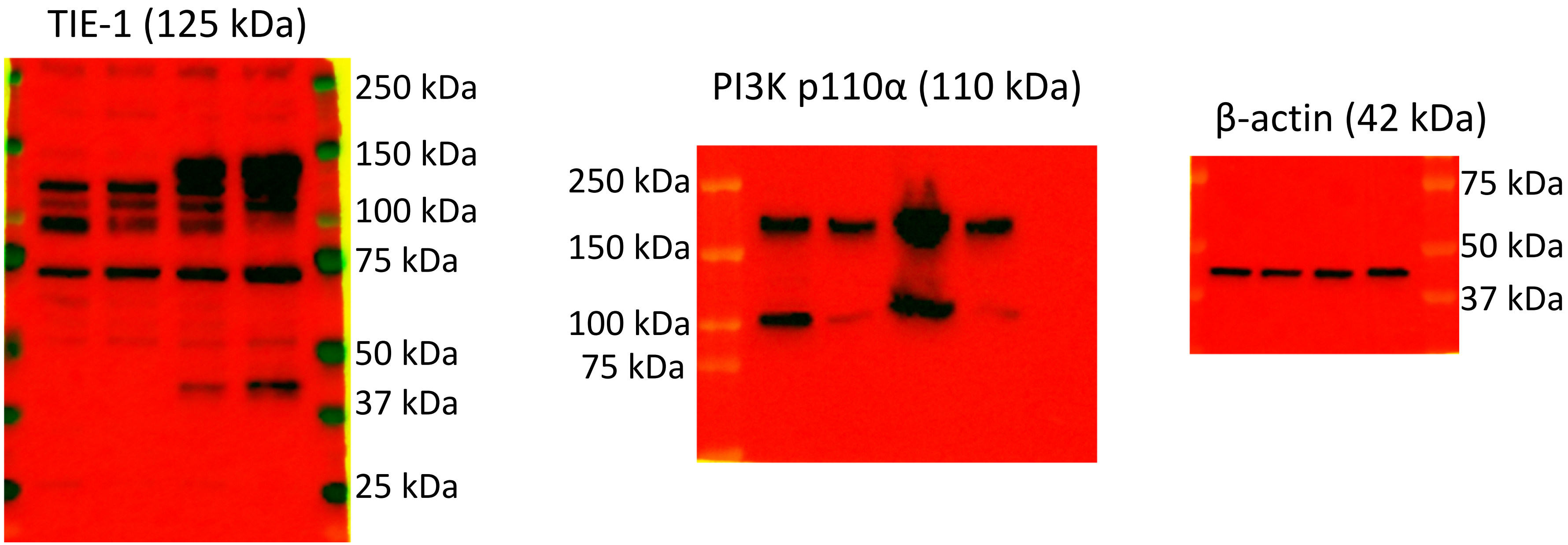


| densitometry readings | Empty-control siRNA | Empty-PI3K siRNA | TIE-1-control siRNA | TIE-1-PI3K siRNA |
|---|---|---|---|---|
| TIE-1 | 70.1 | 84.0 | 180.9 | 231.9 |
| PI3K p110α | 51.7 | 6.6 | 96.9 | 6.2 |
| β-actin | 27.1 | 26.7 | 27.8 | 33.0 |

Supplemental Figure 1

p-PI3K p85 (85 kDa)
p-PI3K p55 (60 kDa)

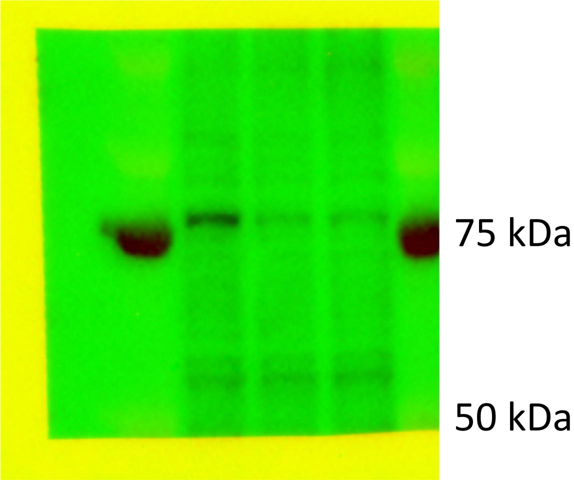


AKT (60 kDa)

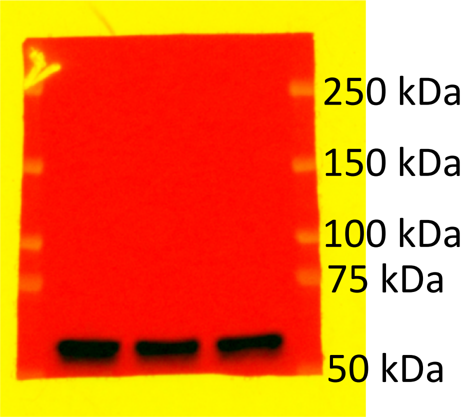


| densitometry readings | Control siRNA | TIE-1 siRNA-1 | TIE-1 siRNA-2 |
|---|---|---|---|
| p-PI3K p85 | 32.8 | 16.9 | 18.6 |
| p-PI3K p55 | 23.2 | 21.4 | 22.8 |
| AKT | 77.9 | 67.6 | 68.3 |

p-PI3K p85 (85 kDa)
p-PI3K p55 (60 kDa)

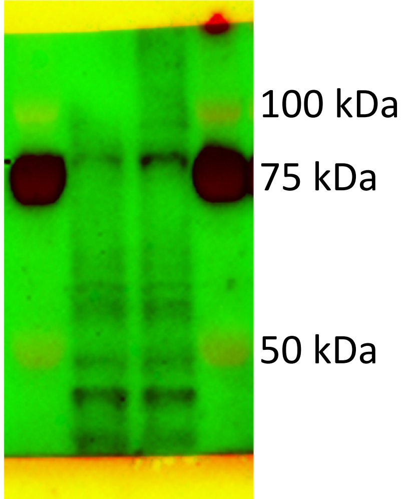


AKT (60 kDa)

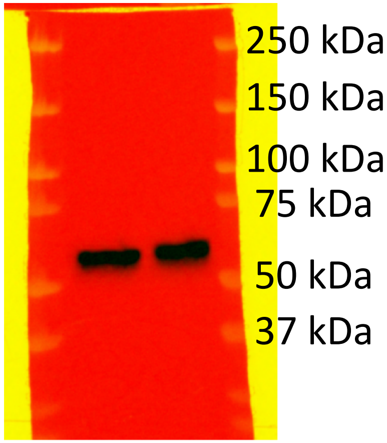


| densitometry readings | Empty vector | TIE-1-V5 vector |
|---|---|---|
| p-PI3K p85 | 33.7 | 47.3 |
| p-PI3K p55 | 36.7 | 36.6 |
| AKT | 73.9 | 71.7 |

Supplemental Figure 4

Procaspase-3 (35 kDa)

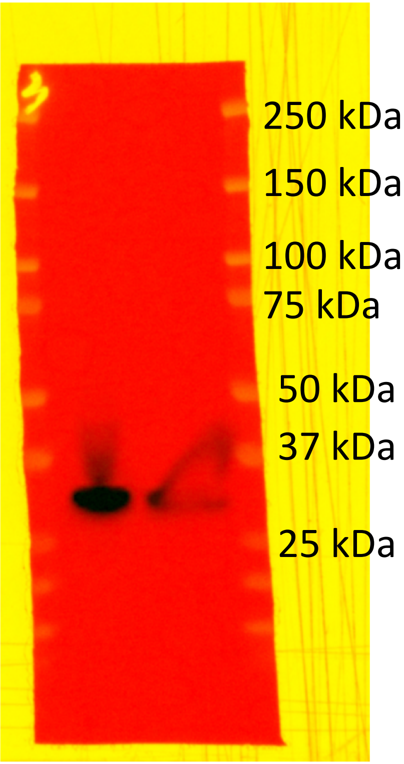


PARP (116 kDa)
Cleaved PARP (89 kDa)

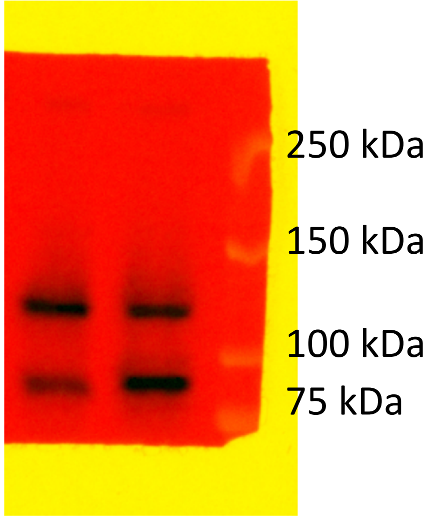


β-actin (42 kDa)

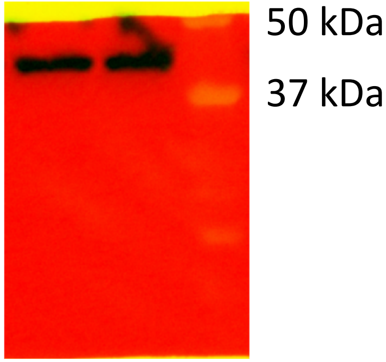


| densitometry readings | CAOV3 control siRNA | CAOV3 TIE-1 siRNA |
|---|---|---|
| PARP | 47.3 | 41.5 |
| Cleaved PARP | 28.2 | 51.0 |
| Procaspase-3 | 82.5 | 31.2 |
| β-actin | 61.8 | 65.2 |

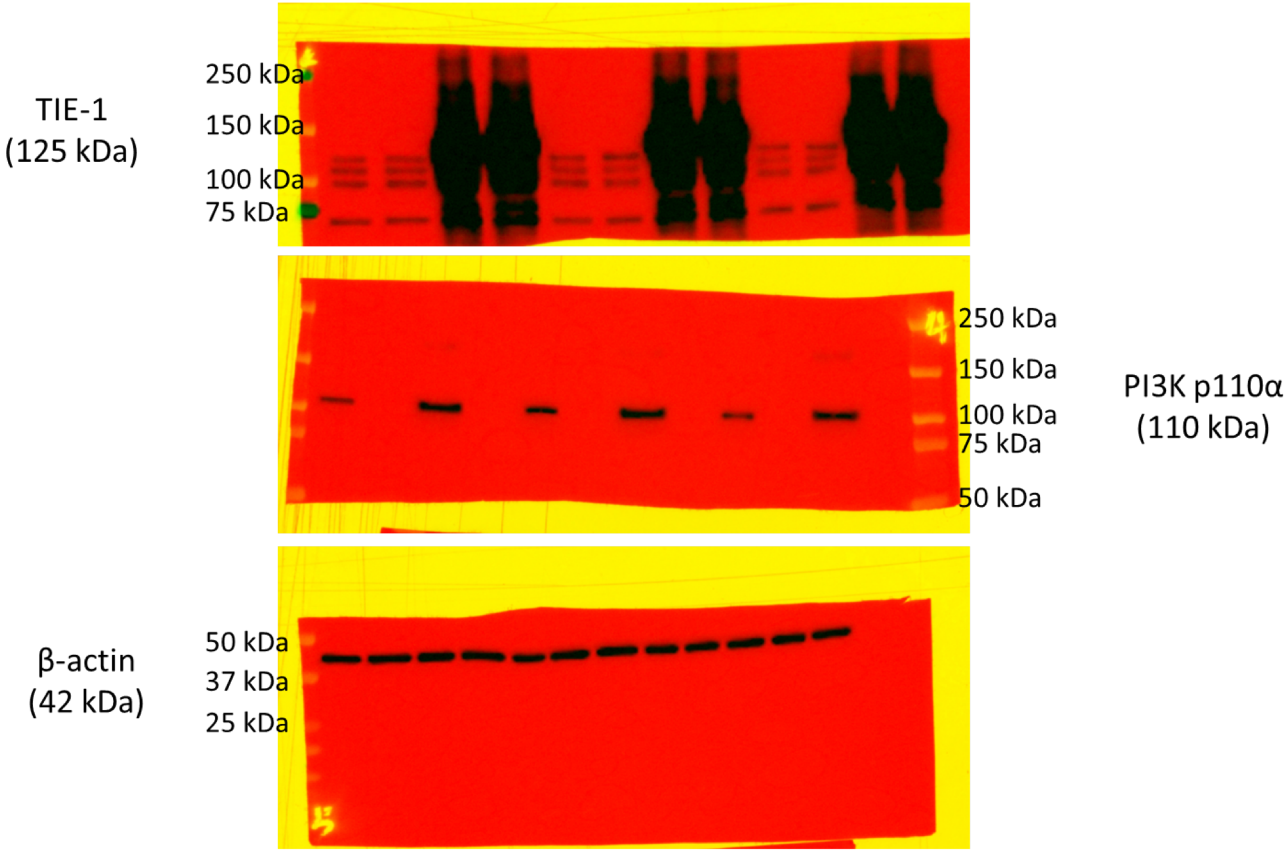


| densitometry readings | Day 3 **Empty** Control si | Day 3 **Empty** PI3K si | Day 3 **TIE-1 OE** Control si | Day 3 **TIE-1 OE** PI3K si | Day 5 **Empty** Control si | Day 5 **Empty** PI3K si | Day 5 **TIE-1 OE** Control si | Day 5 **TIE-1 OE** PI3K si | Day 7 **Empty** Control si | Day 7 **Empty** PI3K si | Day 7 **TIE-1 OE** Control si | Day 7 **TIE-1 OE** PI3K si |
|---|---|---|---|---|---|---|---|---|---|---|---|---|
| TIE-1 | 27.4 | 37.7 | 268.4 | 272.9 | 35.5 | 37.5 | 266.1 | 220.7 | 28.3 | 34.6 | 227.9 | 270.9 |
| PI3K p110α | 15.3 | 1.1 | 53.1 | 1.2 | 29.6 | 1.0 | 58.3 | 1.3 | 19.5 | 1.0 | 50.6 | 1.2 |
| β-actin | 42.8 | 49.1 | 48.5 | 51.4 | 37.7 | 48.3 | 54.6 | 42.8 | 42.1 | 46.9 | 44.0 | 45.7 |

TIE-1 (125 kDa)

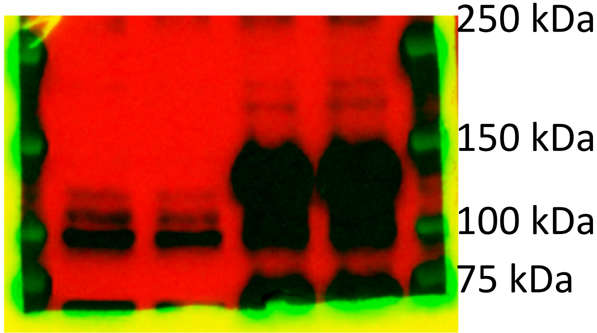

250 kDa
150 kDa
100 kDa
75 kDa

PI3K p110α (110 kDa)

250 kDa
150 kDa
100 kDa
75 kDa

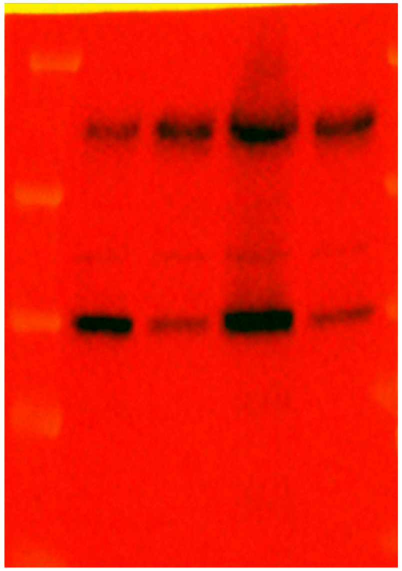

β-actin (42 kDa)

50 kDa
37 kDa
25 kDa

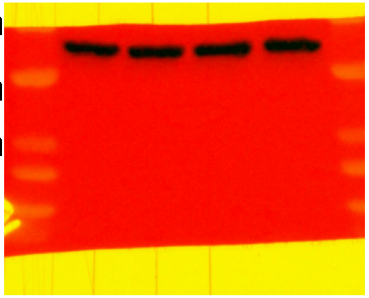

| densitometry readings | Empty-control siRNA | Empty-PI3K siRNA | TIE-1-control siRNA | TIE-1-PI3K siRNA |
|---|---|---|---|---|
| TIE-1 | 67.8 | 49.9 | 258.3 | 285.8 |
| PI3K p110α | 11.8 | 3.4 | 21.7 | 3.8 |
| β-actin | 34.8 | 35.4 | 38.3 | 37.5 |
